# Draft Genome Sequence of *Sphingobacterium* sp. Strain PM2-P1-29, a Tetracycline-Degrading TetX-Expressing Aerobic Bacterium Isolated from Agricultural Soil

**DOI:** 10.1128/genomeA.00963-14

**Published:** 2014-10-09

**Authors:** Sudeshna Ghosh, Timothy M. LaPara, Michael J. Sadowsky

**Affiliations:** a Biotechnology Institute, University of Minnesota, St. Paul, Minnesota, USA; b Department of Civil, Environmental, and Geo- Engineering, University of Minnesota, Minneapolis, Minnesota, USA; c Department of Soil, Water, and Climate, University of Minnesota, St. Paul, Minnesota, USA

## Abstract

The genome of *Sphingobacterium* sp. strain PM2-P1-29 was sequenced. The bacterium contains a physiologically active *tet*(X) gene, encoding a tetracycline-degrading monooxygenase. To our knowledge, this is the only bacterium naturally harboring *tet*(X) for which tetracycline degradation has been demonstrated.

## GENOME ANNOUNCEMENT

The *Sphingobacterium* genus belongs to the *Bacteroidetes* phylum, previously regarded as part of the *Cytophaga*-*Flavobacterium*-*Bacteroides* group. The members of this genus are widely distributed in soil, plants, and animal guts. We sequenced and assembled the genome of *Sphingobacterium* sp. strain PM2-P1-29, isolated from soil near the pig pen of a small farm (1). The genomic sequences of 6 other members of the *Sphingobacterium* genus are available; among them, *Sphingobacterium spiritivorum* ATCC 33300 and ATCC 33861 (2), both clinical isolates, have the closest 16S rRNA gene sequence identity (93.5%) to PM2-P1-29. Strain PM2-P1-29, however, has a greater 16S rRNA gene sequence identity (98.5%) to *Sphingobacterium faecium* NBCR 15299^T^, isolated from cattle feces, whose genome is not available.

PM2-P1-29 contains the *tet*(X) gene encoding monooxygenase-degrading tetracycline, and it transforms tetracycline *in vivo* (3). The *tet*(X) gene is widely detected in the environment and commensal *Bacteroides* spp., where it is nonfunctional (4, 5). In recent years, *tet*(X) has been reported in a duck pathogen (6) and in diverse clinical isolates (7). The TetX enzyme can transform all known tetracyclines, including tigecycline; thus, the increasing prevalence of *tet*(X) is of great concern (8, 9). To our knowledge, PM2-P1-29 remains the only bacterium harboring *tet*(X) with demonstrated TetX activity in its native host. The sequencing of the genome of this bacterium was carried out with the objective of learning more about its genomic organization and the presence of other antibiotic resistance genes.

PM2-P1-29 was grown aerobically at 30°C in Luria-Bertani growth medium (containing 10 g/liter tryptone, 5 g/liter yeast extract, 10 g/liter NaCl). The cells were harvested after overnight growth, and DNA was extracted using the DNeasy blood and tissue kit (Qiagen, Venlo, the Netherlands). Genomic DNA was submitted to the University of Minnesota Genomics Center (UMGC) (Minneapolis, MN) for library preparation and Illumina MiSeq 2 × 250 bp sequencing. DNA from PM2-P1-29 was multiplexed with genomic DNA from 23 other bacterial isolates. The raw reads were trimmed at UMGC to remove library tags and bases of <Q30. The sequence ends were removed to obtain 225-bp sequences, and they were further trimmed for quality using Mothur (version 1.29.2), allowing for 0 ambiguous bases, a maximum of 8 homopolymers, and a minimum quality score of 35 over a rolling window size of 50 nucleotides (10). The trimmed sequences were assembled using Velvet (version 1.2.09), with a *k*-mer length of 31 and a minimum contig length of 1,500 bp (11). The median coverage depth was ~32×. Redundant contigs were identified using CD-HIT (12) and were removed.

The contigs were annotated by Genoscope (13). The number of contigs in the draft genome is 189, with the largest contig containing about 4.1 Mb of DNA. The total length of the genome is 5.4 Mb. PM2-P1-29 has two copies of the *tet*(X) gene, both on a putative mobile element, and it contains numerous regions characteristic of mobile genetic elements. While PM2-P1-29 is resistant to several antibiotics and contains multiple predicted resistance genes, the only other known resistance gene is *aadS* (3).

### Nucleotide sequence accession numbers.

The draft whole-genome sequence has been submitted to the European Nucleotide Archive (ENA) under accession numbers LK931673 to LK931861 and is mirrored in GenBank.
